# Genetically diverse mouse platform to xenograft cancer cells

**DOI:** 10.1242/dmm.049457

**Published:** 2022-08-29

**Authors:** Jennifer K. Sargent, Mark A. Warner, Benjamin E. Low, William H. Schott, Todd Hoffert, David Coleman, Xing Yi Woo, Todd Sheridan, Sonia Erattupuzha, Philipp P. Henrich, Vivek M. Philip, Jeffrey H. Chuang, Michael V. Wiles, Muneer G. Hasham

**Affiliations:** 1 The Jackson Laboratory for Mouse Genetics, 600 Main Street, Bar Harbor, ME 04609, USA; 2 The Jackson Laboratory for Genomic Medicine, 10 Discovery Drive, Farmington, CT 06032, USA; 3 Hartford Hospital, Department of Pathology, 80 Seymour Street, Hartford, CT 06102, USA

**Keywords:** Mouse models, Genetic diversity, Xenograft, Cancer

## Abstract

The lack of genetically diverse preclinical animal models in basic biology and efficacy testing has been cited as a potential cause of failure in clinical trials. We developed and characterized five diverse RAG1 null mouse strains as models that allow xenografts to grow. In these strains, we characterized the growth of breast cancer, leukemia and glioma cell lines. We found a wide range of growth characteristics that were far more dependent on strain than tumor type. For the breast cancer cell line, we characterized the spectrum of xenograft/tumor growth at structural, histological, cellular and molecular levels across each strain, and found that each strain captures unique structural components of the stroma. Furthermore, we showed that the increase in tumor-infiltrating myeloid CD45^+^ cells and the amount of circulating cytokine IL-6 and chemokine KC (also known as CXCL1) is associated with a higher tumor size in different strains*.* This resource is available to study established human xenografts, as well as difficult-to-xenograft tumors and growth of hematopoietic stems cells, and to decipher the role of myeloid cells in the development of spontaneous cancers.

## Abstract (precis)

**Editor's choice:** This article describes the structural, cellular and molecular characterization of five mouse models that allow the grafting of human cancers.

## INTRODUCTION

Xenografting human cancer cell lines or tumor fragments in mice is an established biological system to generate mouse models to study the *in vivo* behavior of the cancer and to test the efficacy of chemotherapeutic compounds (Okada et al., 2019; Rygaard and Povlsen, 1969; Uthamanthil et al., 2017; Yoshida, 2020). In addition, xenografting human hematopoietic stem cells in mice has been a key component in ‘humanizing’ the mouse to study the interaction of the human immune system with cancers and to develop immunotherapies (De La Rochere et al., 2018; Shultz et al., 2012). Human tumors that have been successfully passaged in mice are known as patient-derived xenografts (PDXs), and libraries of these samples are currently used to advance cancer research and anti-cancer therapy (Shi et al., 2020; Uthamanthil et al., 2017; Yao et al., 2019). It is well established that tumors engrafted and passaged in mice maintain a high degree of molecular fidelity compared to the original patient tumors (Woo et al., 2021). Indeed, up to 87% of anti-cancer agent responses in PDX models have directly translated to the drug's efficacy in the actual patient who provided the tumor sample (Izumchenko et al., 2017). Cancer cell lines and PDX models are thus suitable for preclinical drug testing and are key to personalized approaches to cancer treatment.

To xenograft human tissues or cells, mice must be immunodeficient, lacking at least B- and T-lymphocytes (Uthamanthil et al., 2017). Currently, the choice strains for such studies are the inbred immunodeficient NOD/ShiLtJ SCID IL-2R gamma knockout (NSG™) and the NOD/ShiLtJ *Rag1^−/−^* and IL-2R gamma double knockout (NRG) mice (Maletzki et al., 2020; Pearson et al., 2008; Shultz et al., 2014; Uthamanthil et al., 2017). The NSG mouse has a SCID mutation that disrupts multiple immune cell functions, and the *Rag1^−/−^* mice cannot perform V(D)J recombination, leading to the arrest of B- and T-cells in the bone marrow (Fugmann, 2001; Sandhu et al., 1996). These mutations on the strain NOD/ShiLtJ, combined with the deletion of IL-2 receptor gamma enhancing the immunodeficiency, have produced mouse models that can accept xenografts (Fan et al., 2004; Serreze and Leiter, 1994; Shultz et al., 2005). In addition, there are pseudo-outbred strains that can accept foreign tissue. These are the Crl:CD1 (CD-1) nude mice or other athymic nude mice (Rygaard and Povlsen, 1969; Sharkey and Fogh, 1984; Szadvari et al., 2016).

The tumor microenvironment is essential for the growth of a neoplastic cell and determines the therapeutic response (Hinshaw and Shevde, 2019; Wu and Dai, 2017). With just the NOD/ShiLtJ and CD-1 strains, the microenvironment is genetically limited. This led to the National Institutes of Health (NIH), in June 2021, to cite a lack of genetic diversity in preclinical studies as one of the main reasons for failure of clinical trials (ACD Working Group on Enhancing Rigor, Transparency, and Translatability in Animal Research; https://acd.od.nih.gov/documents/presentations/06112021_RR-AR%20Report.pdf). Other organizations in Europe have developed guidelines that also require multiple mouse strains to validate scientific claims (https://arriveguidelines.org; https://norecopa.no/prepare). Hence, there is a need for generating more genetically diverse strains that can accept xenografts.

To establish a platform for characterizing the biology of these cancerous cells *in vivo* and for testing novel therapies in the context of genetic diversity, we characterized tumor growth in a panel of *Rag1^−/−^* genetically diverse mouse strains. Because these *Rag1^−/−^* strains have no B- or T-lymphocytes, this allows cancerous cells to establish and grow *in vivo* in different strains (Fugmann, 2001). The strains reported in this study are genetically diverse based on their single-nucleotide polymorphism profiles and, moreover, are the parental lines of the Collaborative Cross (CC)/Diversity Outbred (DO) mice and the current mouse populations that mimic the genetic diversity in humans (Gralinski et al., 2015; Li and Auwerx, 2020; Petkov et al., 2004; Svenson et al., 2007; Wei et al., 2020). Here, we present the development of a five-strain panel of mice that can accept different cell lines and describe the diverse nature of the resulting xenografted tumors and the host milieu that is associated with tumor growth. This five-strain panel can now be used as the first step in understanding the contribution of genetic diversity in the tumor microenvironment when studying a cancer or establishing a novel therapy.

## RESULTS

### Establishing genetically diverse *Rag1^−/−^* mouse strains

The parental strains of the CC and DO panels include five classical inbred strains (A/J, C57BL/6J, 129S1/SvImJ, NOD/ShiLtJ, NZO/H1LtJ) and three wild-derived strains (CAST/EiJ, PWK/PhJ and WSB/EiJ). These strains capture, on average, 90% of the known allelic diversity across the mouse genome and were thus targeted for *Rag1* knockout (Chesler et al., 2008; Roberts et al., 2007). Three *Rag1^−/−^* strains are readily available at The Jackson Laboratory, including two CC/DO parental strains [NOD/ShiLtJ *Rag1^−/−^* (JR003729) and C57BL/6J *Rag1^−/−^* (JR002216)] and BALB/cJ *Rag1^−/−^* (JR003145). Because the BALB/cJ *Rag1^−/−^* mice are readily available, their addition increases the genetic diversity of our panel; thus, we included this strain in our studies.

We targeted the rest of the CC/DO parental strains (129S1/SvImJ, A/J, CAST/EiJ, NZO/HiltJ, PWK/PhJ and WSB/EiJ) for *Rag1* inactivation using a CRISPR/Cas9 Base Editor system to convert a glutamine codon in *Rag1* (Q192) to a stop codon, resulting in an early termination allele predicted to knock out the gene (Fig. S1A). A second site (Q456) was targeted simultaneously as a substitute in case the first site failed. Although both guides generated multiple founders, the specific substrains selected to establish the knockout strains remained wild type at this second target site. With this method, an additional six strains were obtained. Three strains (129S1/SvImJ, A/J and WSB/EiJ) had the correct *Rag1* mutation, and the remaining three strains produced deletions rather than this particular base change [CAST/EiJ (1 bp DEL), NZO/HiltJ (14 bp DEL) and PWK/PhJ (379 bp DEL)]. Ultimately, all would produce *Rag1* nullizygosity. *Rag1* knockout in WSB/EiJ could not be established due to low fecundity (Table S1), which has been associated with RAG1 deficiency (Aleman et al., 2001; Kekalainen et al., 2015). The PWK/PhJ, CAST/EiJ and NZO/HiltJ mice exhibit complex phenotypes requiring deeper characterization beyond the scope of this study and are thus the subject of a separate investigation.

Hence, our panel of five genetically diverse *Rag1*-deficient mice in this study includes 129S1/SvImJ *Rag1^−/−^* (129R), A/J *Rag1^−/−^* (A/JR), BALB/cJ *Rag1^−/−^* (BAR), C57BL/6J *Rag1^−/−^* (B6R) and NOD/ShiLtJ *Rag1^−/−^* (NR). To confirm the absence of B- and T-lymphocytes in these mice, blood from *Rag1^+/+^* and *Rag1^−/−^* mice from each strain was stained for CD45, a leukocyte marker, CD19, a B-lymphocyte marker, and TCR beta (also known as TRBC1), a T-lymphocyte marker. As shown by flow cytometry, both B- and T-lymphocytes were absent in all five *Rag1* mutant strains, but a fraction of CD45 was present, indicating an intact myeloid compartment, because CD45^+^ cells encompass both the adaptive (B- and T-lymphocyte) and the myeloid immune system (Fig. S1B).

### Genetic background influences xenograft tumor growth

**Fig. 1.**
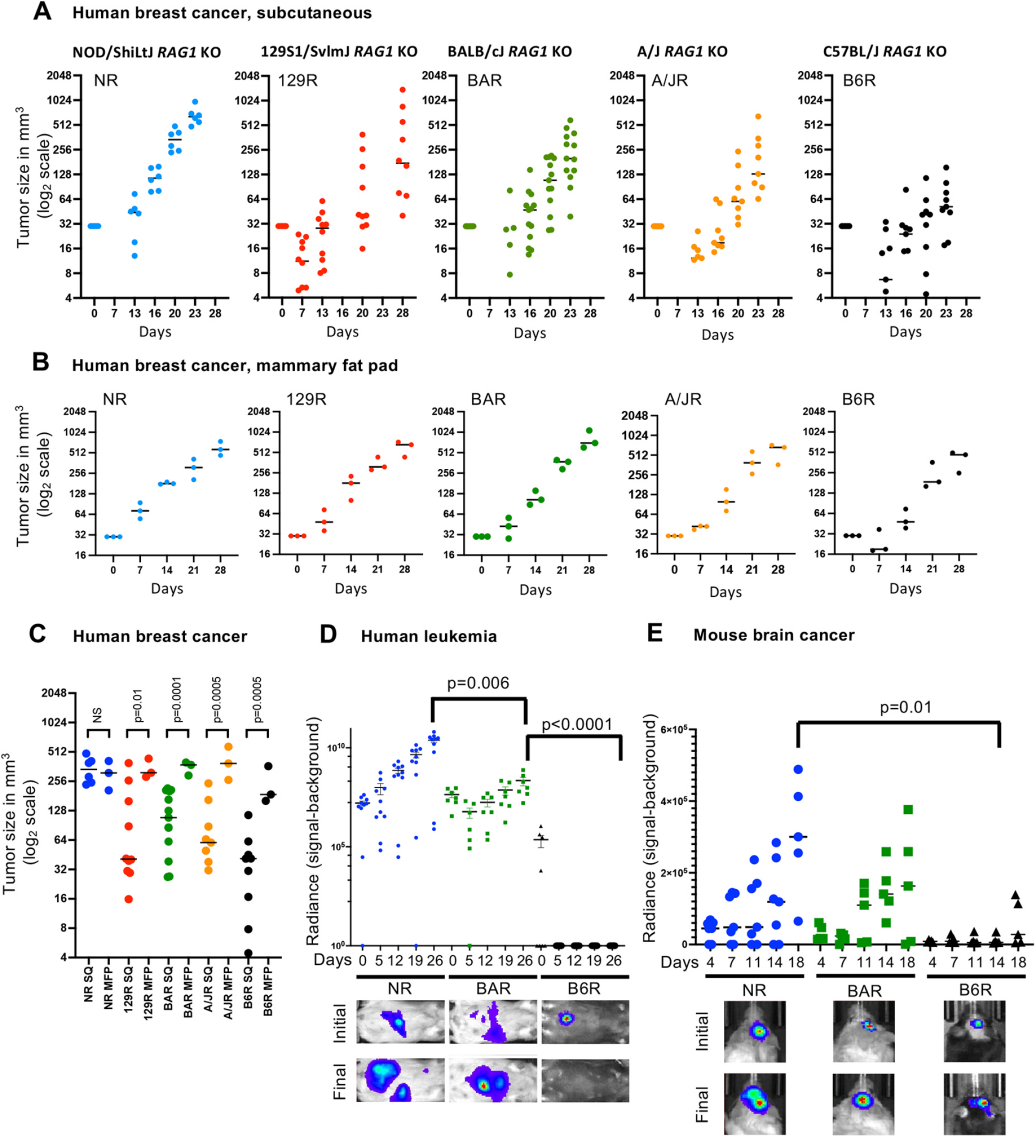
**Growth of xenografts depends on the host mouse strain.** (A) MDA-MB-231 cells were xenografted subcutaneously into the five strains, and the tumor sizes were measured for 4 weeks. The data shown are from pooled male and female mice from three experiments. (B) MDA-MB-231 cells were xenografted in the mammary fat pad and measured for 4 weeks. (C) Comparative size at Day 21 between the subcutaneous and mammary fat pad growth of MDA-MB-231 cells in each strain. MFP, mammary fat pad; NS, not significant; SQ, subcutaneous. (D) Representative images and graph showing bioluminescence of MEC1 leukemia cell line and engraftment showing growth in the three strains studied (data pooled from two experiments). (E) Representative images and graph showing bioluminescence of GL261 glioma cell line and engraftment showing growth in all three strains. For all cell lines and xenografts in this figure, each dot represents one mouse, the bar shows the median, and the *P*-value is calculated by unpaired, two tailed Student’s *t*-test.

To validate the ability of these strains to support the growth of human or mouse grafts in genetically diverse mouse models, we tested the engraftment of (1) MDA-MB-231, a human triple-negative breast cancer cell line; (2) MEC1, a human chronic lymphocytic leukemia cell line; and (3) GL261, a murine glioma cell line. For the breast cancer cell line, both heterotropic and orthotopic xenografts were performed. For the leukemia and glioma cell lines, orthotopic xenografts were used (Fig. 1).

The MDA-MB-231 cell line was xenografted both orthotopically and subcutaneously, and all tumors were measured with calipers. Although the key metric for xenografting is either acceptance or rejection, we observed that the MDA-MB-231 cells successfully xenografted in all these strains. However, the subcutaneously implanted tumors had different growth rates in each strain (Fig. 1A). This suggests that the genetic background of the strain plays a far greater role in tumor progression than previously thought, because the same amount and type of cells were xenografted in each mouse. The NR strain showed the largest tumor size after 4 weeks, similar to that of control NSG^TM^ and NRG mice (Fig. S2A). The next strain that showed large tumor growth was 129R, followed by BAR, A/JR and finally B6R. As a panel, these five strains model a complete spectrum of xenograft tumor growth for the MDA-MB-231 human triple-negative breast cancer cell line.

Because MDA-MB-231 is a breast cancer, we also xenografted the cells orthotopically in the mammary fat pad of each strain to test how the cells grew in their own natural microenvironment (Fig. 1B). An interesting pattern emerged. Orthotopically, there was no difference in the growth between strains after 4 weeks, unlike that observed in the cells subcutaneously xenografted. When subcutaneous and orthotopic xenografts from each strain were compared against each other at the 3-week point, a time point that is commonly used to test tumor viability [beyond this time tumors start developing ulcerations or core necrosis (>500 mm^3^)], there was no significant difference in the NR strain. However, 129R, BAR, A/JR and B6R showed a significant difference between the two methods (Fig. 1C).

We further wanted to test whether the growth pattern observed with the solid tumor in different strains can be recapitulated with other types of cancers and chose to test the growth of two other cell lines, human leukemia (MEC1) and murine glioma (GL261). For these experiments, we chose the mouse strains that supported the highest (NR), intermediate (BAR) and lowest (B6R) growth of subcutaneously engrafted MDA-MB-231 cells. Both MEC1 and GL261 cell lines host a luciferase transgene that is activated with luciferin and can be imaged to quantify the extent of the orthotopic tumor growth *in vivo* via bioluminescence signal. A growth pattern emerged that was similar to that of the subcutaneously xenografted MDA-MB-231 human breast cancer cells, with NR supporting the most extensive tumor growth, BAR intermediate and B6R the least, for MEC1 cells (Fig. 1D). The GL261 cell line was developed in the C57BL/6 strain (Ausman et al., 1970). Therefore, it was expected that an orthotopic syngeneic allograft should be favored in the B6R mice. Surprisingly, NR and BAR were able to support more extensive growth of GL261 cells compared to the syngeneic B6R host. Together, these findings suggest that the genetically diverse tumor microenvironment contributes more to the cancer growth than does the neoplasticity or the origin of the cancer cell (Fig. 1E).

A notable feature observed is that there were no significant differences in the growth of MDA-MB-231 cancer cells between NSG, NRG and NR mice. IL-2 receptor gamma was knocked out in NOD/ShiLtJ in combination with either *Rag1* knockout or with the SCID mutation, which is necessary to reduce myeloid function (Brehm et al., 2014; Maletzki et al., 2020; Okada et al., 2019; Pearson et al., 2008; Shultz et al., 2014; Shultz et al., 2005). However, with respect to these xenografts, the absence of IL-2 receptor gamma seems to have had no significant effect (Fig. S2A).

### Genetic background influences histological features of xenograft tumor growth

The aforementioned results suggest that tumors can grow in different immunodeficient strains and therefore can be used in novel compound efficacy testing. Before these strains are used for efficacy testing, there needs to be a comprehensive analysis of the structural, cellular and molecular milieu for each of these strains. To perform such analysis that has clinical significance and reproducibility in other laboratories, we opted to use the standard method of xenografting (subcutaneous implantation) of a commonly used breast cancer cell line, MDA-MB-231, to expound on the differences between the strains.

**Fig. 2.**
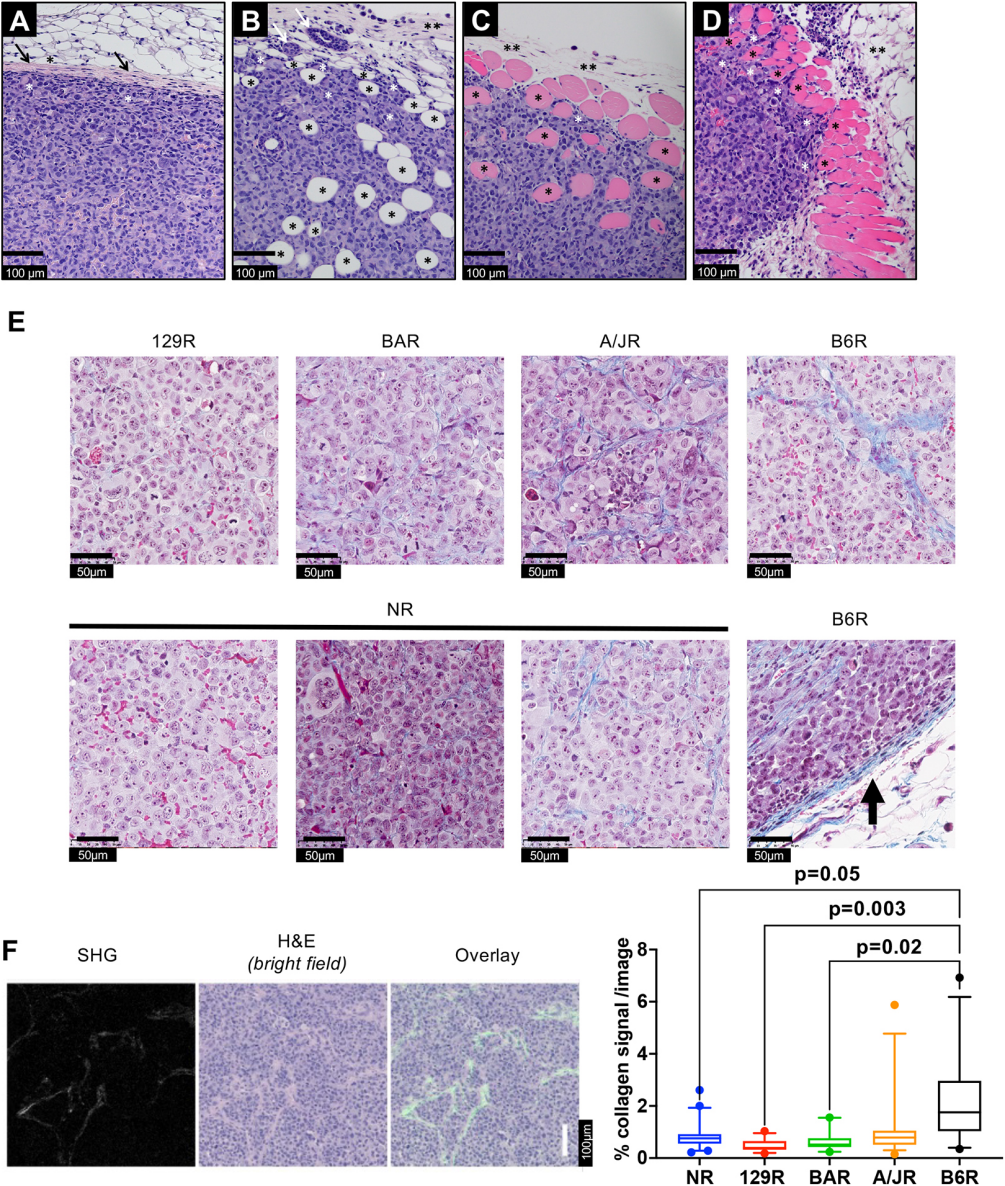
**Representative images of masses composed of xenografted neoplastic cells illustrating variation in growth patterns.** (A) B6R mouse with an encapsulated expansile mass. A layer of mouse fibrous tissue forming a capsule (black arrows) surrounds and separates the implanted neoplastic cells (white asterisks) from mouse subcutaneous tissue (adipocytes, black asterisk). (B) 129R mouse with an unencapsulated infiltrative mass. Implanted neoplastic cells infiltrate into adjacent mouse subcutaneous tissue resulting in adipocytes (black asterisks) at the mass margin becoming surrounded by neoplastic cells (white asterisks). Mouse fibrovascular connective tissue (fascia) is indicated by a black double asterisk. (C) A/JR mouse with an unencapsulated infiltrative mass. Implanted neoplastic cells infiltrate into adjacent mouse skeletal muscle tissue resulting in myofibers (black asterisks), which stain pink, at the mass margin becoming surrounded by neoplastic cells (white asterisks). Pre-existing mouse fibrovascular connective tissue (fascia) is indicated by black double asterisks. (D) NR mouse with an unencapsulated infiltrative mass. Implanted neoplastic cells infiltrate into adjacent mouse skeletal muscle tissue resulting in myofibers (black asterisks) at the mass margin becoming surrounded by neoplastic cells (white asterisks). Mouse subcutaneous tissue (adipocytes) is indicated by a black double asterisk. (E) Masson trichrome staining showing the differential amounts of collagen in different strains responding to the same cell line. Masses in B6R have additional collagen around the tumor (black arrow), and masses in NR have all three types of collagen density within the same tumor. (F) Second-harmonic generation (SHG) collagen signal (left) showing the original, 8-bit signal collected by the 2P-M instrument setup. Center panel shows brightfield image generated via pathology slide-scanning instrument. The right panel shows the overlay of SHG images, with a false-color representation (green) for better visibility on top of the corresponding area of the histopathology image. Boxes show 5-95% confidence interval with the median, whiskers indicate 95% confidence interval. 20-41 images from three biological replicates from each strain, illustrating the percentage collagen signal per image analyzed. *P*-values calculated by nested one-way ANOVA (Tukey) with multiple comparisons. Non-significant comparisons are not shown.

In general, the growth patterns and supporting stroma characteristics of the tumor masses differed among host mouse strains (Fig. 2). In all strains, implanted neoplastic cells formed variably sized, round to irregularly shaped masses composed of anaplastic neoplastic epithelial cells. The medium-to-large-sized polygonal neoplastic epithelial cells often lacked distinct borders and showed small-to-moderate amounts of eosinophilic finely granular-to-lacy cytoplasm and a medium-to-large-oval-to-indented-to-irregular nucleus. Nuclei had a reticular-to-finely granular chromatin pattern with one or two medium-sized magenta nucleoli. Many large-to-giant multinucleated neoplastic cells were noted.

Strain-specific differences in tumor structure were also notably observed with regard to collagen abundance and structure. In general, larger tumors were associated with less collagen fiber, both in thickness and general network complexity (Fig. 2). Specifically, tumor masses in B6R mice, which support the smallest tumors, were encapsulated by several layers of fibrous tissue that compressed the surrounding tissues (Fig. 2A). Tumor masses in 129R (Fig. 2B) and A/JR (Fig. 2C) mice, which host intermediate-sized tumors, showed unencapsulated masses with neoplastic cells that infiltrated into surrounding mouse tissues, including fat or muscle or both (myofibrils are pink and fat is clear). Tumor masses in NR mice (Fig. 2D), which support the largest tumors, were also unencapsulated but were less infiltrative relative to masses in 129R and A/JR mice.

To confirm the aforementioned observation with regard to the amount of collagen observed, the tumors were analyzed for collagen by Masson trichrome staining. Although all tumor masses were composed of closely packed epithelial cells that form small solid nests within a fibrovascular stroma composed of blood vessels, the collagen network within tumor masses varied among mouse strains (Fig. 2E). 129R showed the least amount of infiltrative collagen staining, followed by BAR, A/JR and B6R. Masses in 129R and BAR mice demonstrated very thin fibrovascular stroma containing scant amounts of collagen fibers, whereas masses in B6R mice consisted of notably thick strands of multiple collagen fibers. Masses in NR mice showed variable amounts of collagen from very thin to moderately thick within the same tumor, but always less collagen relative to masses in B6R mice. To quantitate the collagen amounts, we used a second-harmonic generation (SHG) imaging system (Campagnola, 2011; Lichtman and Conchello, 2005). With this system, it was observed that B6R showed a significantly higher amount of collagen compared to NR, 129R and BAR strains. The amounts of collagen differed between A/JR and B6R, albeit with a *P*-value of 0.08 (Fig. 2F; Fig. S2B). It is therefore possible that the limited tumor growth in B6R mice is related to a strain-specific difference in the collagen network encapsulating the neoplastic cells.

### Genetic background influences the proportion of neoplastic cells in the tumor

**Fig. 3.**
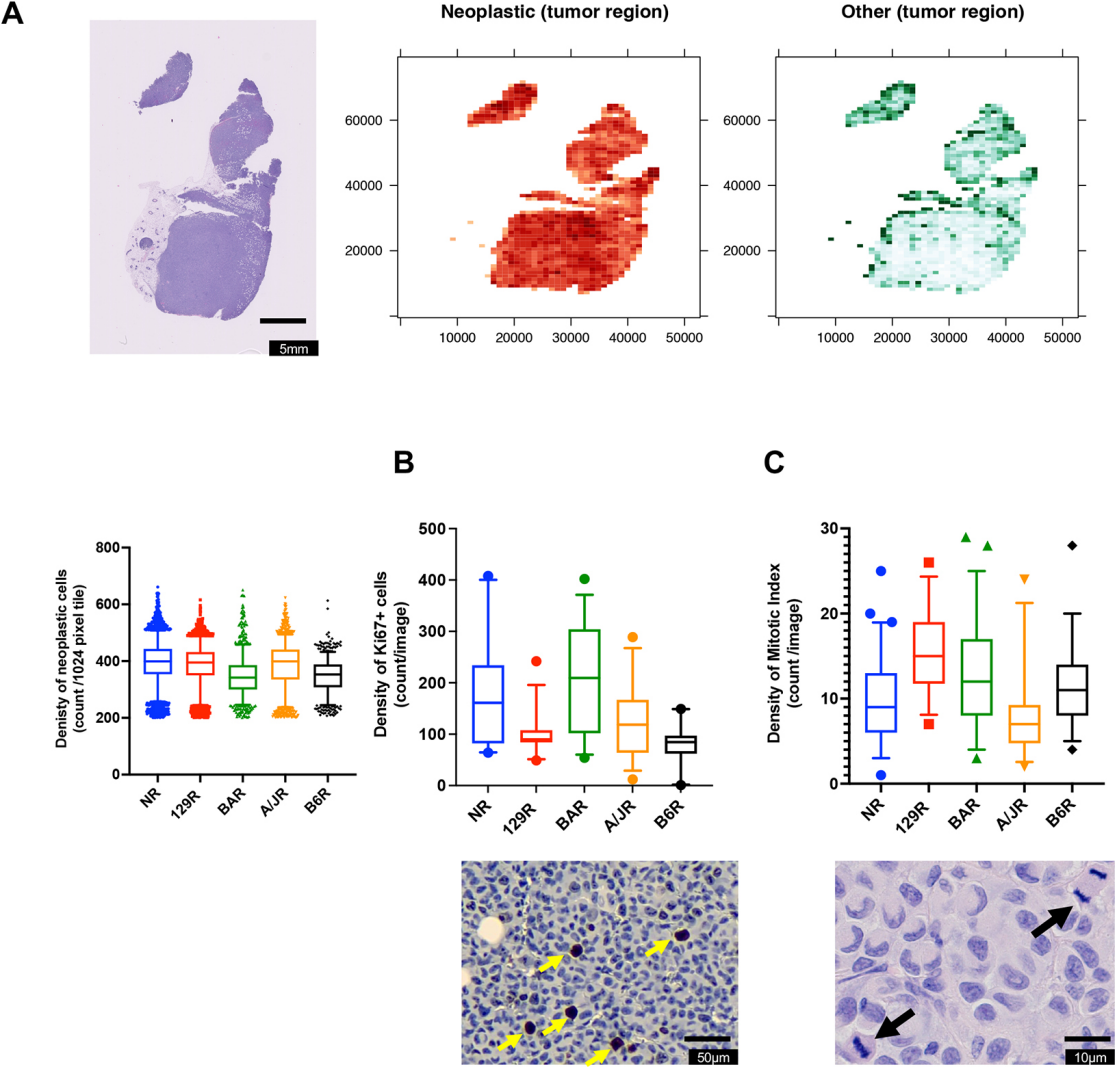
**Tumor cell content from whole-slide images of *Rag1^−/−^* strains.** (A) Representative H&E image (left), spatial distribution of neoplastic nuclei density (center), and spatial distribution of all other nuclei types combined (connective, inflammatory, non-neoplastic epithelial, dead and non-labeled; right) for each strain. The nuclei density is defined as the number of nuclei per 1024×1024 pixel tile. The heatmaps are restricted to tumor regions with >200 neoplastic nuclei per tile. In the box and whisker plot below, boxes show 5-95% confidence interval with the median, whiskers indicate 95% confidence interval (849-3506 images from three biological replicates from each strain). (B) Representative image of Ki67 staining (yellow arrows indicate a positive cell) with the box and whisker plot (boxes show 5-95% confidence interval with the median, whiskers indicate 95% confidence interval; 18-30 images from three biological replicates from each strain) for density of Ki67-positive cells. (C) Representative image of mitotic cells (black arrows) with the box and whisker plot (boxes show 5-95% confidence interval with the median, whiskers indicate 95% confidence interval; 30-60 images from three to six biological replicates from each strain) for density of mitotic index. *P*-values by nested one-way ANOVA accounting for multiple measurements per tumor. The statistical differences found comparing the strains are indicated in the text.

We employed an advanced computational imaging approach using HoVer-Net to define the cell types in tumor masses in each of the mouse strains xenografted with MDA-MB-231 cells (Fig. 3). HoVer-Net employs a convolutional neural network for simultaneous segmentation and classification of nuclei in whole-cell images. To validate the effectiveness of HoVer-Net in distinguishing neoplastic from other (connective, immune, necrosis and non-neoplastic epithelial) cell types in our tumors, Hematoxylin and Eosin (H&E) images of tumors from an initial cohort comprising B6R, BAR, NRG and NSG strains (tumor samples from three different mice per strain) were evaluated. Selected tiles with a total of 27,982 segmented nuclei were assessed by a board-certified pathologist and compared to HoVer-Net predictions (Fig. S3A). We observed an average accuracy of 0.83, precision of 0.78, recall of 0.92 and F1-score of 0.84 across the four strains in terms of classifying neoplastic nuclei (Fig. S3B). Thus, HoVer-Net allows us to quantitatively compare the cellular composition of the tumors in the different strains.

We then applied HoVer-Net to images of tumors from these strains, with tumor slide samples from three different animals per strain. Fig. S4A shows example tiles overlayed with nuclei segmentation and classification for each strain. The computational approach to segmentation and classification of nuclei by HoVer-Net enables us to quantify the total and spatial distribution of nuclei types for the entire whole-slide image (Fig. 3A; Fig. S4B and Fig. S5). The tumor mass in all strains was dominated by spatially variable but a high density of neoplastic nuclei, with a low density of other nuclei (Fig. S4B). A higher density of other nuclei was observed at the periphery of the tumor, suggesting higher stromal infiltration. The contrast between the peripheral and the central tumor region was most apparent for the B6R strain, which hosts the smallest tumors. This is consistent with the pathologist's assessment that the tumors in the B6R strains were encapsulated by fibrous mouse tissue (Fig. 2E,F). The distribution of local neoplastic and other nuclei counts per tumor is shown in Fig. S4B, in which variability within and between tumors of each strain is apparent. Fig. 3 compares the combined spatial heterogeneity in density of the cell types between the strains.

However, large proportions of the tumors have low counts of other nuclei across all strains (median range 21-42 nuclei per tile), with very limited regions displaying high density of these cells. We observed that the overall neoplastic nuclei density based on local absolute counts is higher in the tumors of the NR, 129R and A/JR strains (median of 399, 395 and 399, respectively, versus median of 353 and 342 for B6R and BAR, respectively), while the tumors of the A/JR and 129R strains displayed slightly higher density of other nuclei types (median of 42 and 35, respectively, versus median of 23, 21 and 32 for NR, B6R and BAR, respectively), consistent with the higher extent of stromal infiltration described above.

The neoplastic and other nuclei fraction per tumor, which characterizes the overall mixture of neoplastic and other cells, is shown in Fig. 3A and gives an indication of overall tumor–stromal infiltration per strain. We found that the tumors in the NR strain, which was earlier observed to have the largest tumors and lower degree of infiltration into surrounding tissues, had the highest density and proportion of neoplastic cells. Tumors of the A/JR strain showed highest density of both neoplastic and non-neoplastic cells but had the highest degree of stromal infiltration based on cell fraction. Therefore, the variable spatial cell-level observations across the different strains suggest that the crosstalk between neoplastic cells and the surrounding stromal cells may play a greater role in tumor progression than previously thought.

Neoplastic cells are highly proliferative, and a well-established marker for neoplastic cells is Ki67 (also known as MKI67) positivity (Li et al., 2015). We stained the tumors for Ki67 and observed the amounts of neoplastic cells in NR, A/R and B6R to be similar to those observed in the HoVer-Net analysis (Fig. 3B; Fig. S6). However, 129R and BAR showed a different amount to that observed in the HoVer-Net analysis. Cells that proliferate should also have a higher mitotic index; in fact, there is a correlation between Ki67 positivity and mitotic index (Rudolph et al., 1998). Conversely, NR, a strain with the largest tumors, had the lowest mitotic index (Fig. 3B,C). These three assays measure neoplastic cells in the tumor, and we expected a concordance between them. However, each strain showed a distinct pattern.

Statistically, when a direct ANOVA was used to compare the differences between the strains, all three assays showed a significant difference among the means (*P*<0.0001), if one considers that multiple images of the same tumor capture a different ratio of neoplastic:stroma cells as shown in Fig. 3A. However, if a nested one-way ANOVA was performed accounting for multiple measurements, then only differences between these strains were observed in Ki67 staining, suggesting that the strain determines the propensity of proliferation of a neoplastic cell (*P*=0.0137, *F*=5.435, DFn=4, Dfd=10). These data suggest that the tumor microenvironment of each strain, and not the neoplastic cell, determines the rate of proliferation and the exit towards mitosis.

### Infiltration of myeloid cells correlates with tumor growth in most strains

**Fig. 4.**
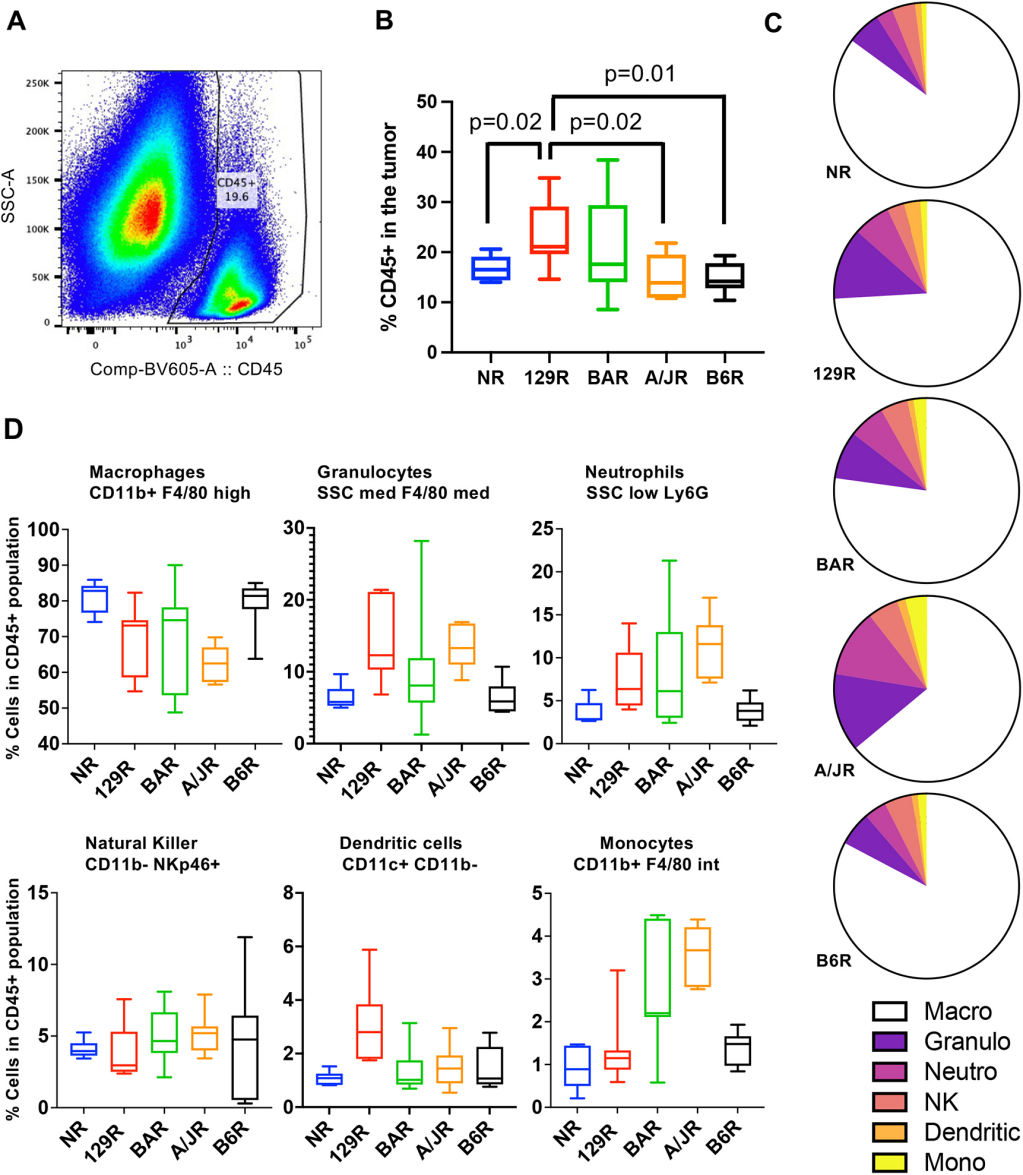
**Tumor-infiltrating myeloid cells positively correlate with tumor size.** (A) Representative flow cytometer gating used to detect CD45^+^ cells (white blood cells) in the tumor. (B) Proportion of CD45^+^ cells in the tumor in *Rag1^−/−^* mice of different strains. Box and whisker plot (boxes show 5-95% confidence interval with the median, whiskers indicate 95% confidence interval; *N*=7 biological replicates from each strain) showing the percentage of CD45^+^ cells in the tumor. Only pairwise statistically significant values are shown. (C) Pie charts showing cell type distribution with the CD45^+^ population. Macro, macrophages; Granulo, granulocytes; Neutro, neutrophils; NK, natural killer cells; Dendritic, dendritic cells; Mono, monocytes. (D) Distribution of different myeloid populations within the CD45^+^ pool from each strain. Boxes show 5-95% confidence interval with the median, whiskers indicate 95% confidence interval (*N*=7 biological replicates from each strain). Statistical analysis of each myeloid population for each strain is shown in Fig. S7.

Although *Rag1^−/−^* strains are deficient in B- and T-lymphocytes, the myeloid immune system is unaffected and could thus attack the tumors (Fugmann, 2001). Therefore, it is important to characterize the remaining immune cells – the myeloid component in the tumors. We examined the number of infiltrating myeloid cells in the tumors from MDA-MB-231 subcutaneously xenografted from each strain by staining for CD45, a leukocyte marker (Fig. 4A). We found that 129R had a significantly higher percentage of CD45^+^ cells than A/JR, B6R and NR, but was not different from BAR (Fig. 4B). It has been shown that NOD/ShiLtJ mice have defective CD45^+^ cells. Therefore, if one removes that strain from the analysis, it is observed that the larger the tumor, the higher the percentage of CD45^+^ cell infiltration (Fig. 4B), but whether the CD45^+^ cells in the tumor are supporting the tumor growth or are bystanders is being currently investigated.

We further characterized the infiltrating CD45^+^ cells to determine whether there was a particular population that predominates within the tumor across the strains. This characterization would allow us to pinpoint whether a certain mechanism is involved in slowing or accelerating the tumor growth (Fig. 4C,D; Fig. S7). We analyzed the CD45^+^ subsets within each strain (Fig. 4C) and across strains (Fig. 4D). Abundance of no specific immune populations appeared to correlate with observed differences in growth in each strain. Interestingly, NR (large tumor sizes) in comparison to B6R (smallest tumor sizes) presents a similar CD45^+^ subset distribution. The expectation was that natural killer (NK) cells would be a major contributor to size because tumors are attacked by these immune cells (Minetto et al., 2019; Vivier et al., 2011). However, neither NK cells nor any subpopulation of myeloid cells was responsible for the tumor size, suggesting that each strain may have their own diverse patterns of myeloid populations infiltrating the tumor.

### Pro-inflammatory cytokines in blood correlate with tumor growth patterns across strains

**Fig. 5.**
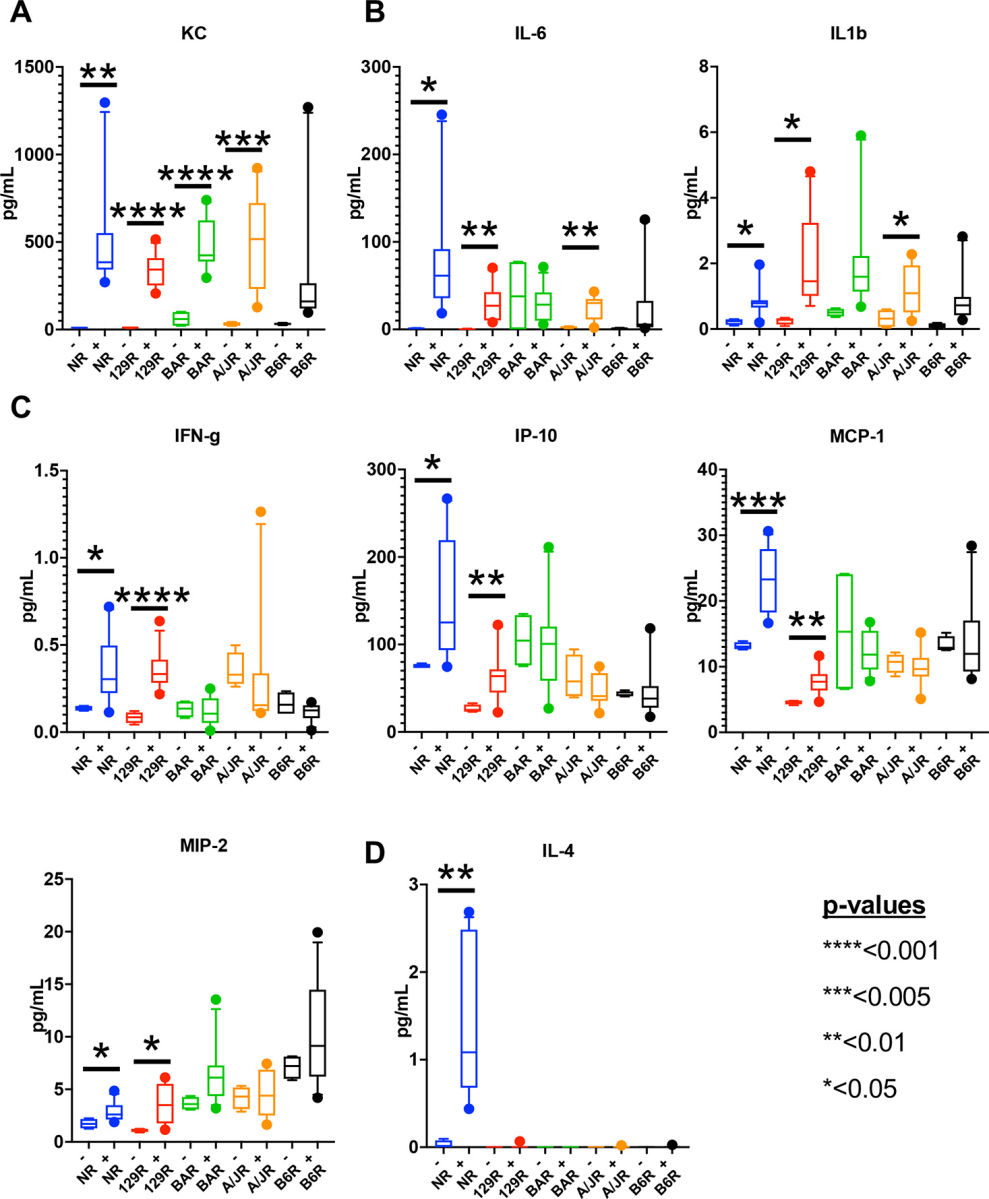
**Circulating levels of pro-inflammatory cytokines and chemokines associate with tumor size.** (A-D) Amount of KC (A); IL-6 and IL-1B (B); IFN-G, IP-10, MCP-1 and MIP2 (C); and IL-4 (D) detected in the plasma of different strains in pg/ml. Boxes show 5-95% confidence interval with the median, whiskers indicate 95% confidence interval. *n*=4-12 samples from three biological replicates from each strain; *P*-values calculated by unpaired, two tailed Student’s *t*-test are shown as asterisks, and values are indicated on the figure. Non-statistically significant differences are not shown.

Pro-inflammatory cytokines and chemokines are drivers of tumor growth and are a major method by which the neoplastic cell and stroma communicate (Candido and Hagemann, 2013; Shurin et al., 2006). To define strain differences in pro-inflammatory cytokine and/or chemokine response to xenograft growth across strains, we interrogated the plasma of mice undergoing the MDA-MB-231 subcutaneous xenografting using the Meso Scale 29-plex assay system. This allowed the comparison of 29 cytokines and chemokines found in the plasma between xenografted and unxenografted control mice after 4 weeks (Fig. 5). When the amounts of cytokines in the plasma of the xenografted mice from each strain were compared to those in the plasma of non-xenografted control mice from the same strain, we observed that keratinocyte-derived chemokine (KC; also known as CXCL1) protein was significantly increased in all but one strain (B6R) (Fig. 5A). KC is a pro-inflammatory chemokine associated with cancer growth and tumor-associated macrophages that modify the tumor microenvironment (Van Damme et al., 2004). In three strains (NR, 129R and A/JR), two other pro-inflammatory cytokines, IL-6 and IL-1B, were observed to significantly increase (Fig. 5B). IL-6, which shows the highest increase of >70-fold in xenografted versus the control plasma, has been shown to promote a microenvironment conducive to tumor growth (Hirano, 2021; Taniguchi and Karin, 2014). We further observed other pro-inflammatory cytokines and chemokines [IFN-G, IP10 (also known as CXCL10), MCP-1 (also known as CCL2) and MIP2 (also known as CXCL2)] to significantly elevate, however, only in NR and 129R, the strains that showed the highest tumor growth (Fig. 5C). Only NR showed an increase in an anti-inflammatory cytokine, IL-4 (Fig. 5D). A large number of chemokines and cytokines either did not show a >2-fold increase [e.g. TNF-A (also known as TNF), IL-16 and MIP3A (also known as CCL2)], significant difference between xenografted and non-xenografted mice [e.g. MIP1A (also known as CCL3), IL-5, IL-33 and IL-10], or were below detection (see list, Fig. S8). These data support the observation that certain pro-inflammatory cytokines and chemokines, particularly IL-6 (70-fold induction) and KC (30-fold induction), may be associated with strain-specific tumor growth.

## DISCUSSION

In this study, we established a platform for characterizing the biology of cancerous cells *in vivo* and for testing novel therapies in the context of genetically diverse mouse models. In addition, we developed a panel of five genetically diverse host strains that can be used to define a wider array of characteristics of cancers and test drug efficacy than is currently standard practice. We have characterized salient structural, cellular and molecular aspects of the solid subcutaneous tumors within each strain. The most important aspect about this study is that we used the same neoplastic cells across all the host strains, so that we could clearly measure the effect of the host on the host–cancer cell interaction, thus solidifying the importance of genetically diverse models. Here, we show, using our panel of three to five host strains, that the host tumor microenvironment is the key determining factor with respect to growth for these specific breast and brain tumors, and leukemia.

Although the CC and DO strains mimic the human genetic diversity, here we show that our panel of five host strains provides a resource sufficient to capture diversity in breast and brain tumors, and leukemia. As a result, this panel is an economical yet still powerful method for screening xenografts. Although DO mice are the gold standard with regards to mapping alleles, this requires hundreds of costly mice, demanding vast resources of time and space, and, notwithstanding, the mice must be immunodeficient to accept the xenografts. However, it is possible to capture these alleles by simply performing an F1 and, subsequently, F2 crosses of the permissive and resistant host strains bearing the tumor of interest. Such F1/F2 methods have led to the identification of *Sirpa* allele from NOD/ShiLtJ as a key component for xenograft acceptance – when NOD/ShiLtJ *Sirpa* allele was engineered into the very resistant BALB/cJ and C57BL/6J strains, these modified strains could accept and develop human hematopoietic and cancer stem cells (Jinnouchi et al., 2020; Kwong et al., 2014; Strowig et al., 2011).

Neoplastic cells that develop into solid tumors rely on the structure to support the growth of the cancerous cells. In this study, we have observed two phenomena that are host dependent. First, the ability to penetrate the surrounding tissue – the same neoplastic cells could penetrate some host tissues far better than other host tissues, with the more permissive host environment allowing greater penetration and better tumor growth. Second, the more collagen fibers a host strain provides, the less the tumor grows. These two features are specific to the host that allows the neoplastic cell to be able to establish growth. Additionally, the extent of stromal infiltration into the tumor region, which we have quantified by nuclei segmentation and classification using a deep-learning approach, can alter the tumor microenvironment and affect treatment outcomes (Valkenburg et al., 2018). We also noted that, when using these hosts, tumor-infiltrating myeloid cells correlated positively with greater tumor size except for the NR strain, which, as discussed in the Results, is known to have a defective myeloid system. These data corroborate previous studies that show that specific components of the myeloid immune system that facilitate syngeneic tumor growth (Galdiero et al., 2018; Hinshaw and Shevde, 2019; Kim and Bae, 2016). In this paper, we have not delved into the cellular mechanism of proliferation and mitosis as observed by computational or molecular markers such as Ki67 and the size of the tumor. It suffices to say that the two variables are discordant (BAR has the highest Ki67 staining but is smaller than NOD). This suggests that mechanisms other than just proliferation determine the size of the tumor; these could include mechanisms of cell death and/or the composition of the non-neoplastic cells.

Within this panel of host strains, we observe that increase in pro-inflammatory cytokines – such as KC, IL-6, IL-1B, IFN-G, IP-10, MCP-1 and MIP2 – in the plasma from the host is associated with better growth of the xenografted cells. Although we also observed an increase in anti-inflammatory cytokines such as IL-4 and IL-10, the fold increase in pro-inflammatory cytokine such as IL-6 far exceeded the anti-inflammatory cytokine production. Whether the structural, cellular and molecular support from the host is the cause or the effect that promotes the tumor growth cannot be distinguished in this study. We speculate that the growth and the observed structure/cells/molecules influence each other in a feedback loop, and understanding such a molecular feedback loop will lead to novel treatments of cancer.

Cell line-derived xenografts and PDX models have been successfully established for breast, lung, colorectal, pancreatic and many other types of cancers. However, xenograft failure rates due to various biological reasons including host rejection, even in immunocompromised hosts, remains between ∼20% and 100% depending on tumor type (Lai et al., 2017). As an example, there are currently no PDX models for slow-growing oligodendrogliomas. Engraftment failure results in a lost sample and a lost opportunity to obtain clinically relevant data for that patient or tumor type. Perhaps xenografting a diverse panel of mice will increase the probability of tumor establishment.

Overall, here, we report a five-strain resource that will be available to the scientific community to dissect the role of the non-neoplastic tumor microenvironment in the context of genetic diversity. This resource is the first step towards addressing the issue of improving human health across all genetic backgrounds.

## MATERIALS AND METHODS

### Animal use

All protocols and experiments that were performed were approved by The Jackson Laboratory (JAX) Institutional Animal Care and Use Committee (IACUC), and all regulations and accreditations are approved by the American Association for Accreditation of Laboratory Animal Care (AAALAC). As described in the JAX Facilities Document, “Comparative Medicine and Quality (CMQ) advances and protects the health, welfare, and genetic quality of animals at JAX. Animal Welfare and Compliance assures that JAX routinely meets the highest standards of animal care and humane treatment; and coordinates the activities of the IACUC, which oversees all aspects of the care and use of animals at the Laboratory, including training in biomethodology and surgery. JAX Veterinary Services provide clinical veterinary care for the animals, animal care oversight, and surgically altered animals on request.” All mice (*Mus musculus*) were obtained and reproduced at JAX (Bar Harbor, ME, USA). Mice used were between the ages of 8 and 12 weeks of age when xenografted and housed in disposable boxes with alpha-dri pad bedding, kept in a pressurized individual ventilated rack in an Animal Biosafety Level 2 (ABSL2) mouse room space. All breeding units were in a separate non-ABSL2 mouse room location, different from where procedures were performed. All mice had free access to 6% fat sterilized food and acidified water.

### Knocking out *Rag1^−/−^*

To establish genetically diverse immunocompromised host strains of mice, we engineered *Rag1* knockout in selected stains using either CRISPR/Cas9 or Base Editing technology (BE4max) (Koblan et al., 2018), according to the scheme depicted in Fig. S1A. Both methods used the same sgRNA (5′-acagtcaggtctacttccca-3′, Synthego) generating a Q192X early termination modification when coupled with a base editor (BE; Trilink, custom mRNA) or an NHEJ-induced frameshift mutation when used with Cas9. As a backup for the BE-derived mutants, a second sgRNA was included (5′-tcatgcaaggcaggggctcc-3′, Synthego), designed to generate another early termination (Q456X) allele. Delivery of reagent into wild-type zygotes was achieved using either microinjection (BE targeting: 100 ng/µl BE4max mRNA, 50 ng/µl each sgRNA) or electroporation [CRISPR/Cas9 targeting: Cas9 protein (PNA Bio): 250 ng/µl, 100 ng/µl sgRNA]. Mutant alleles were identified and characterized by PCR and sequencing (o2686, 5′-ATCTGTGGGAATCGTTTCAAGA-3′; o2687, 5′-AGAAGGACTTTCTCGGCATTCC-3′). Candidate founders were backcrossed to wild-type cohorts from the host strain at least once before inbreeding was initiated. Homozygotes were subsequently confirmed for the lack of B- and T-lymphocytes (CD19 and TCR beta markers, respectively) using flow cytometry (Fig. S1B).

### Xenografting MDA-MB-231 subcutaneously

Mice were shaved on the flank and given isoflurane (1.5-2%) until anesthetized. Paralube eye ointment (Fisher Scientific, NC1886507) was applied to lubricate the eyes. Mice were moved to a procedure hood where isoflurane anesthesia was maintained via nose cone (1.5-2%). The shaved flank was washed with 70% ethanol followed by chlorhexidine repeated three times. The vial containing cells suspended in Dulbecco's phospho-buffered saline (DPBS) was gently inverted multiple times to evenly distribute the cells and drawn in 100 µl aliquots for subcutaneous xenografts in a 1 ml 26-gauge allergy syringe. Each mouse received 30 mm^3^, which approximates to 2.5±0.5 million cells. After the xenograft, the mouse was moved to a clean box on a heated surface until fully alert. Subcutaneous tumors were measured via calipers. The volume was calculated using (length×width^2^)/2.

### Xenografting MDA-MB-231 orthotopically

Female mice were used to transplant 2.5 million MDA-MB-231 cells in 100 μl DPBS into the mammary fat pad. Mice were anesthetized using isoflurane (1.5-2%), and paralube eye ointment was applied to the cornea to prevent eye drying. The fur on the left side of the abdomen was removed with clippers and then sterilized with 70% ethanol and chlorhexidine. This scrub method was repeated three times. A small 1 cm incision was done medial to the fourth nipple on the right mammary chain. The fat pad was not removed for this procedure, and 100 μl of cells was injected directly into the fat pad using a 22-gauge needle. The incision was closed with a wound clip, and bupivacaine was given topically to the wound. Carprofen was given as an analgesic. Mice were removed from isoflurane and placed onto a heating pad to recover. Once the mice were fully alert, they were placed onto the shelf and monitored daily for 3 days to ensure proper healing. Wound clips were removed after 5 days. Measurements were performed similarly to those for subcutaneous xenografts.

### Xenografting GL261 intracranially

Mice were given isoflurane (1.5-2.5%) until anesthetized, and fur was removed from the head of the mouse using clippers. Carprofen (1 mg/ml) was administered subcutaneously per body weight of animal at 0.1 ml per 10 g. Eye ointment was applied, and the mouse was placed into a stereotactic device. A scalpel was used to make a 2 mm incision above the injection site. A microdrill was used to drill a hole into the skull, and then 2 µl of GL261 cells (10,000 cells) was xenografted into the right hemisphere using a 10 µl syringe (Hamilton, 65460-05). After injection, a sterile cotton swab was used to remove any excess fluids and to dry the skin. Surgilock glue (Fisher Scientific, NC9855218) was applied with an applicator tip to enclose skin together, and 50 µl of 0.1% bupivacaine was applied topically onto the incision. After injections, the mice were placed into a clean box and onto a heated surface until fully recovered from anesthesia.

### Xenografting MEC1 via tail vein

Mice were placed under a heat lamp for 3 min and monitored using a thermometer to ensure proper temperature. A mouse was placed into a clear restraint tube. The tail was cleaned with 70% ethanol, and 10^7^ MEC1 cells were injected via the tail vein with a 26-gauge allergy syringe. Pressure was applied to the injection site to stop any bleeding. After injection, each mouse was placed into a clean box and monitored to ensure good health.

### *In vivo* imaging of mice for bioluminescence

Under isoflurane anesthesia (1.5-2%), mice were injected via intraperitoneal injection with Rediject d- Luciferin (Perkin Elmer, 770504) at 150 mg/kg per mouse. Mice were immediately placed in a Perkin Elmer IVIS Spectrum CT imager under isoflurane anesthesia (1.5-2% administered by nose cone) according to the manufacturer’s instructions. Every sequence run contained a non-xenografted mouse of the same coat color. This ensured a proper background subtraction when analyzing the individual regions of interest (ROIs). ROI boxes were placed around the expected signal regions (full body for leukemia model, skull region for glioma model), and measurements were calculated using Living Image software. The ROI box remained a consistent size throughout the experiments and across all strains to ensure that all data collected were comparable (Fig. 1).

### Immunohistochemistry staining of Ki67 on fixed tumor tissues

Tumors, slides and staining were prepared using a Leica Bond Staining platform and Bond Polymer Efine Detection Kit using citrate for antigen retrieval for 10 min (Leica, DS9800), prepared according to the manufacturer's instructions. Ki67 was prepared and diluted according to the manufacturer's protocol (Thermo Fisher Scientific, RM-9106).

### Fibrillar collagen quantification by SHG imaging

SHG imaging is a powerful and relatively new nonlinear optical imaging modality in which the SHG signals are produced by a non-centrosymmetric nonlinear optical material in response to an intense short laser pulse. Two-photon (2P) excitation of collagen at 800 nm will produce SHG signals at exactly half the wavelength, 400 nm. For this study, we used a Leica SP8 DIVE MP system with a 4Tune detection unit and a tunable ultrafast laser (Insight X3, 680-1300 nm). A Leica 20× oil immersion objective (0.75 HC PL APO CS2) was used to acquire the images. The samples were excited at 800 nm, and the SHG signals were collected at 395-405 nm. Autofluorescence from the tissue is least at this excitation–emission wavelength range, and, by limiting the bandwidth of detection to 10 nm to ∼400 nm, only the SHG signals are detected. Binary threshold operation from the OpenCV library was used to segment the raw SHG images of tumor tissues for collagen fibers from background noise. The ratio of non-zero-pixel values of the binary SHG images to the total pixel area of the images was used for the quantification of the amount of collagen. Overlays of color histopathology images and the SHG signal were generated in Fiji.

### Flow cytometry assay of the tumor

Tumor samples were digested using half the amount of reagent required per sample of Miltenyi Biotech Liver Kit as per the manufacturer's instructions in C-tubes on a gentleMACS™ dissociator (Miltenyi Biotech) with heaters. After digestion, samples were filtered through Falcon filter cap tubes and washed with 2 ml Dulbecco's modified Eagle medium (Gibco). Samples were centrifuged at 500 ***g*** for 5 min, washed twice with AutoMACS Rinsing Solution (Miltenyi Biotech) with 1% bovine serum albumin and 0.5 mM EDTA, and resuspended in 1 ml of the aforementioned solution for counting and viability check on an Attune NxT cytometer. Cells (10^6^) were incubated with the following antibodies: anti-CD16/32 APC-R700, anti-CD11b APC, anti-F4/80 PE-Cy7, anti-EpCAM PerCP-Cy5.5, anti-CD45 BV605, anti-CD11c FITC, anti-NKp46 PE (BD Biosciences; diluted at 1:000 to 1:10.000). Data were acquired on a BD Biosciences FACSymphony A5 cytometer and analyzed with FlowJo Software (BD Biosciences).

### Clinical chemistry of the plasma

Serum was measured for cytokine and chemokine levels on a Meso Scale Discovery MESO QuickPlex SQ 120 instrument. Samples were diluted and processed utilizing a U-PLEX Biomarker Group 1 mouse 29-Plex kit, item number K15355K-1, as per the package insert.

### Nuclei segmentation and classification of H&E images

Histopathological images stained with H&E were obtained for three tumor samples per strain and scanned using a Hamamatsu Nanozoomer scanner. We performed segmentation and classification of nuclei in the H&E images using HoVer-Net (Graham et al., 2019). HoVer-Net (https://github.com/vqdang/hover_net) employs a convolutional neural network for simultaneous nuclear segmentation and classification. We utilized the whole-slide image (WSI) processing option with default parameters and the pretrained dataset PanNuke (arXiv:2003.10778), which labels the nuclei as neoplastic, connective, inflammatory, non-neoplastic epithelial, dead or non-labeled. We validated the HoVer-Net predictions using WSIs from an earlier cohort derived from B6R, BAR, NRG and NSG strains. For this cohort, the segmented and classified nuclei from selected tiles per WSI were assessed by a board-certified pathologist, and wrongly classified nuclei were re-labeled. Selected tiles were chosen to obtain many cases from each class of nuclei. Given the large number of neoplastic nuclei and limited number of nuclei of other classes, we calculated the accuracy, precision, recall and F1-score based on HoVer-Net predictions of neoplastic nuclei versus the other nuclei classes combined. The nuclei density of WSIs was visualized on heatmaps comprising 1024×1024 pixel tiles. When focusing on the tumor regions, we restricted to tiles with >200 neoplastic nuclei per tile, which allowed us to exclude peripheral non-tumor and fat regions. For WSIs with multiple consecutive tissue sections, only one section was used to compute the nuclei density and nuclei proportion.

### Statistics and graphics

Microsoft Word™ and Excel™ version 16.54 (21101001), GraphPad PRISM™ version 9.1.0 (216) and Adobe Acrobat™ Pro DC Version 2021.007.20099 were used to calculate the statistics and generate graphics. Nested one-way ANOVA with multiple comparisons to account for multiple measurements from a tumor was performed to determine the statistical significance of differences between strains. Otherwise, if biological replicates were compared, a direct unpaired, two-tailed Student’s *t*-test was performed. FlowJo™ version 10 was used to analyze flow cytometry data. Fiji (NIH ImageJ2) Version 2.3.0/1.53q Build d544a3f481 was used to quantify the Ki67 images and generate the collagen/H&E overlay images.

## Supplementary Material

Supplementary information
