# Supplementary material for: Genetically diverse mouse platform to xenograft cancer cells
Source: Dis Model Mech. 2022 Aug 29;15(9):dmm049457. doi: 10.1242/dmm.049457 (PMC9459392; doi:10.1242/dmm.049457)
Supplement: Supplementary information [file dmm-15-049457-s1.pdf]

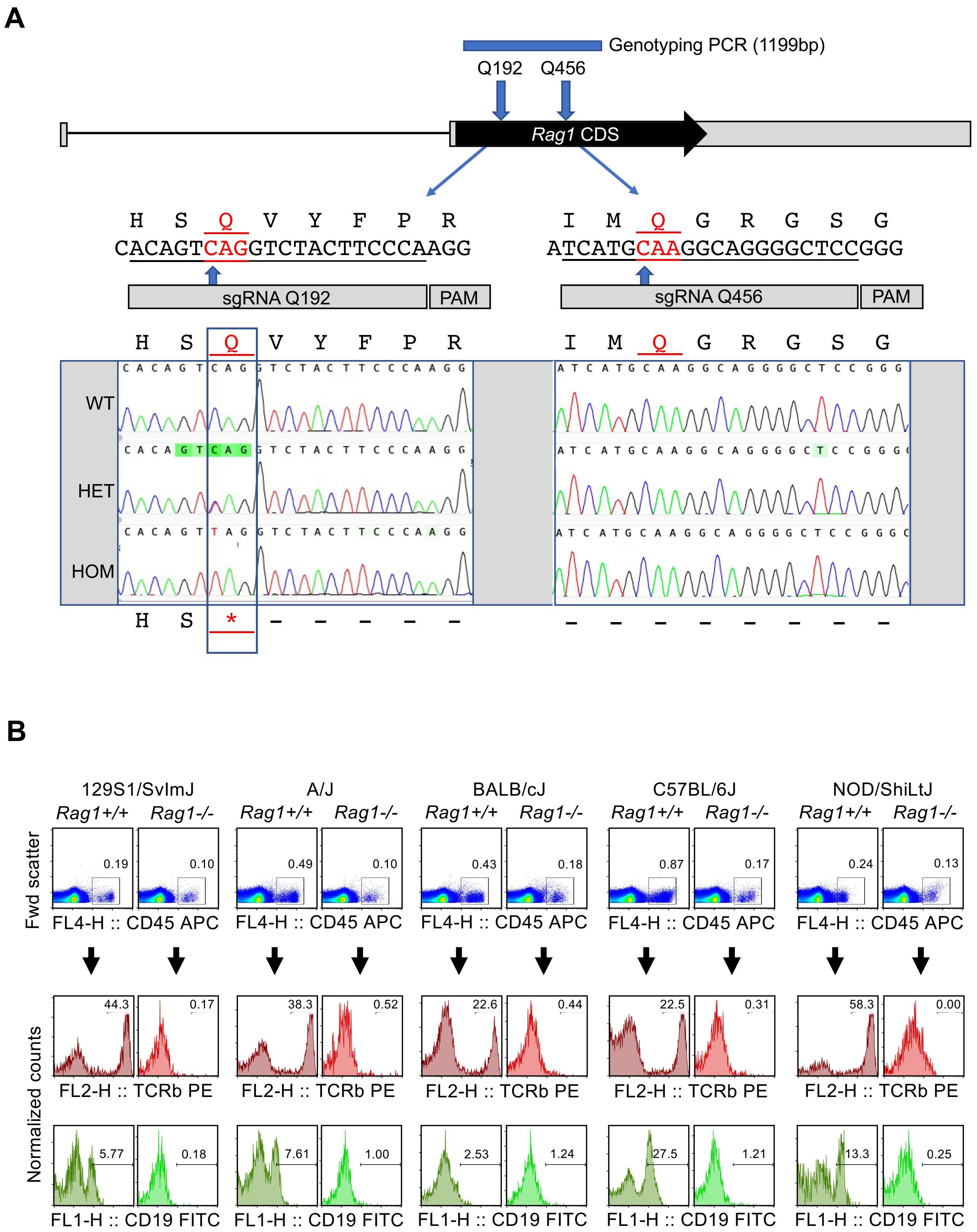

Disease Models & Mechanisms • Supplementary information

Fig. S1. Generation and validation of the *Rag1$^{-/-}$* knock out strains. (A) Schematic of the method used to generate the *Rag1$^{-/-}$* knock out on all the strains except for BAR, B6R, and NR, which were commercially available. (B) Flow Cytometry gating strategy of the blood to validate loss of B- and T-lymphocytes. Flow cytometry example of the CD45 population (all white blood cells) from *Rag1$^{+/+}$* and *Rag1$^{-/-}$* knock out from different strains gated on TCRb (T-lymphocytes) and CD19 (B- lymphocytes) showing complete nullizygosity of the adaptive immune system in the *Rag1$^{-/-}$* strains.

Disease Models & Mechanisms • Supplementary information

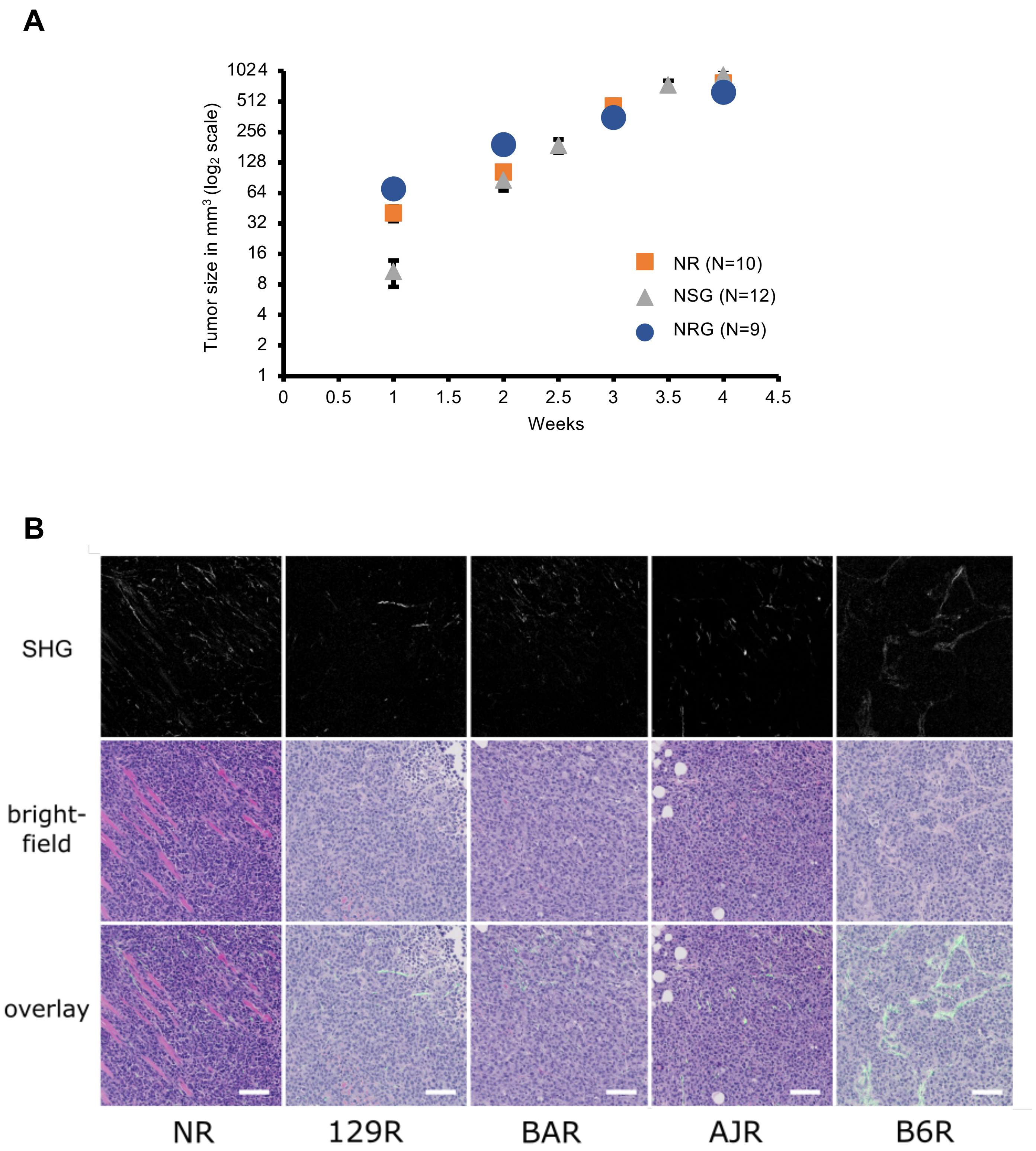

Disease Models & Mechanisms • Supplementary information

Fig. S2. Growth curves of NR, NRG and NSG and collagen staining. (A) Graph showing average growth with standard errors. Data is pooled from 3 experiments. (B) SHG collagen signal (top panels) showing the original, 8-bit signal collected by the 2P-M instrument setup. Center panels show brightfield images generated via pathology slide scanning instrument. The bottom panels show the overlay of SHG images, with a false-color representation (green) for better visibility on top of the corresponding area of the histo-pathology image. Scalebar100µm.

A

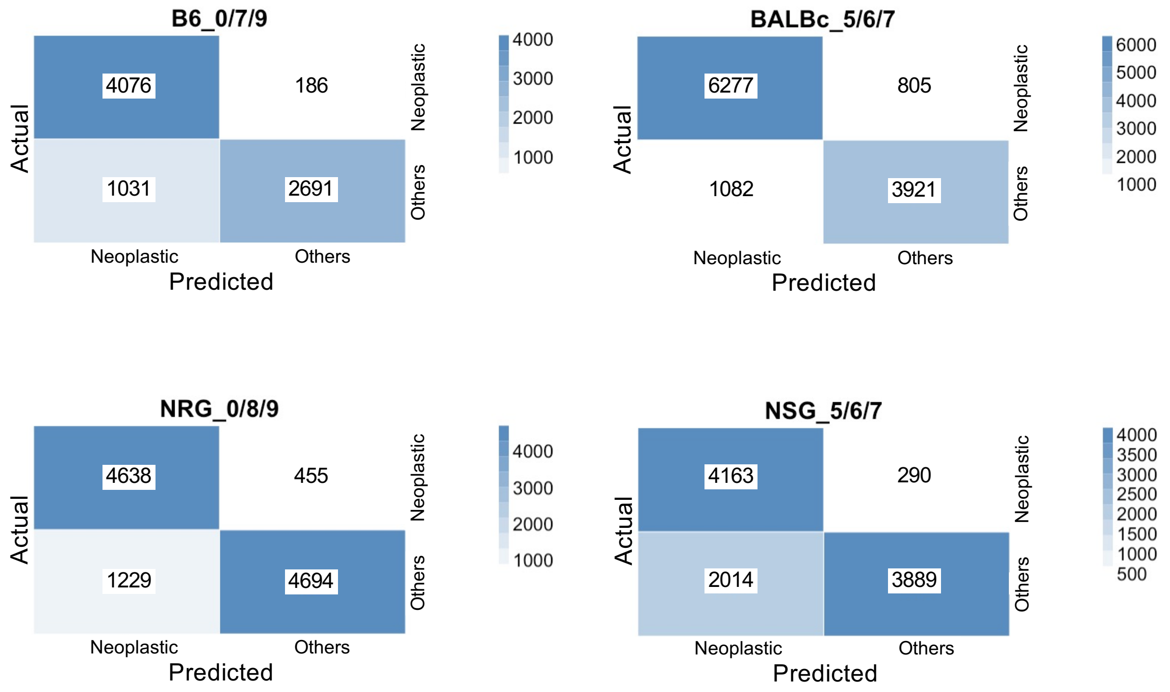

B

Neoplastic vs Others	B6R (n=5479)	BAR (n=8969)	NRG (n=6777)	NSG (n=6757)
Accuracy	0.847	0.843	0.847	0.777
Precision	0.798	0.852	0.790	0.673
Recall	0.956	0.886	0.910	0.934
F1-score	0.870	0.869	0.846	0.783

Fig. S3. Validation of HoVer-Net classifications using B6R, BAR, NRG and NSG strains. (A) Confusion matrices of prediction of neoplastic nuclei by HoVer-Net based on selected tiles from three samples per strain. (B) Accuracy, precision, recall and F1-score for prediction of neoplastic nuclei for each strain.

Disease Models & Mechanisms • Supplementary information

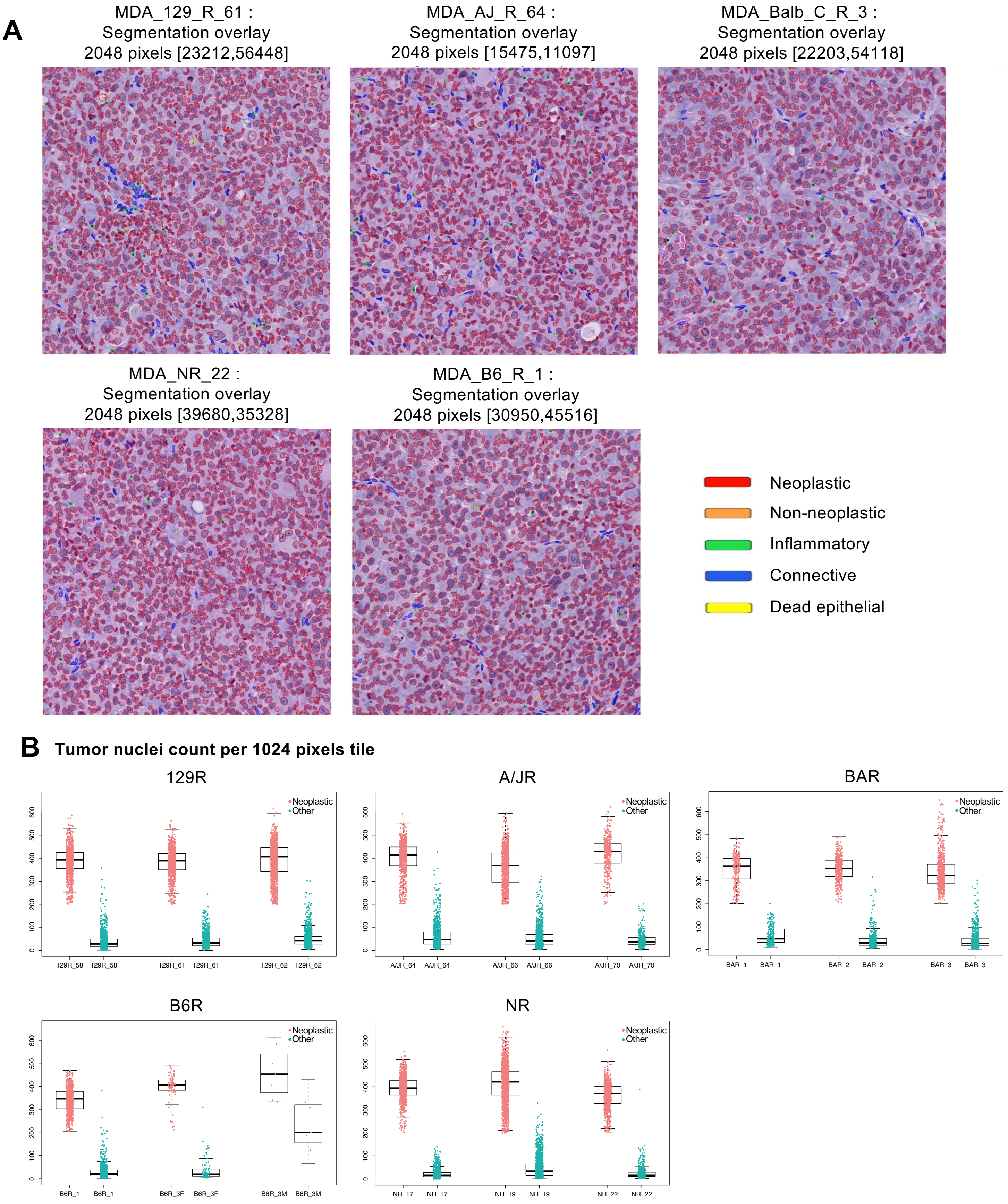

Disease Models & Mechanisms • Supplementary information

Fig. S4. Examples of HoVer-Net segmentation and classification. (A) The nuclei segmentations and classifications are overlaid on 2048 pixel x 2048 pixel regions representative of the H&E images for each strain.(B) Distribution of neoplastic and other nuclei density for each whole slide image.

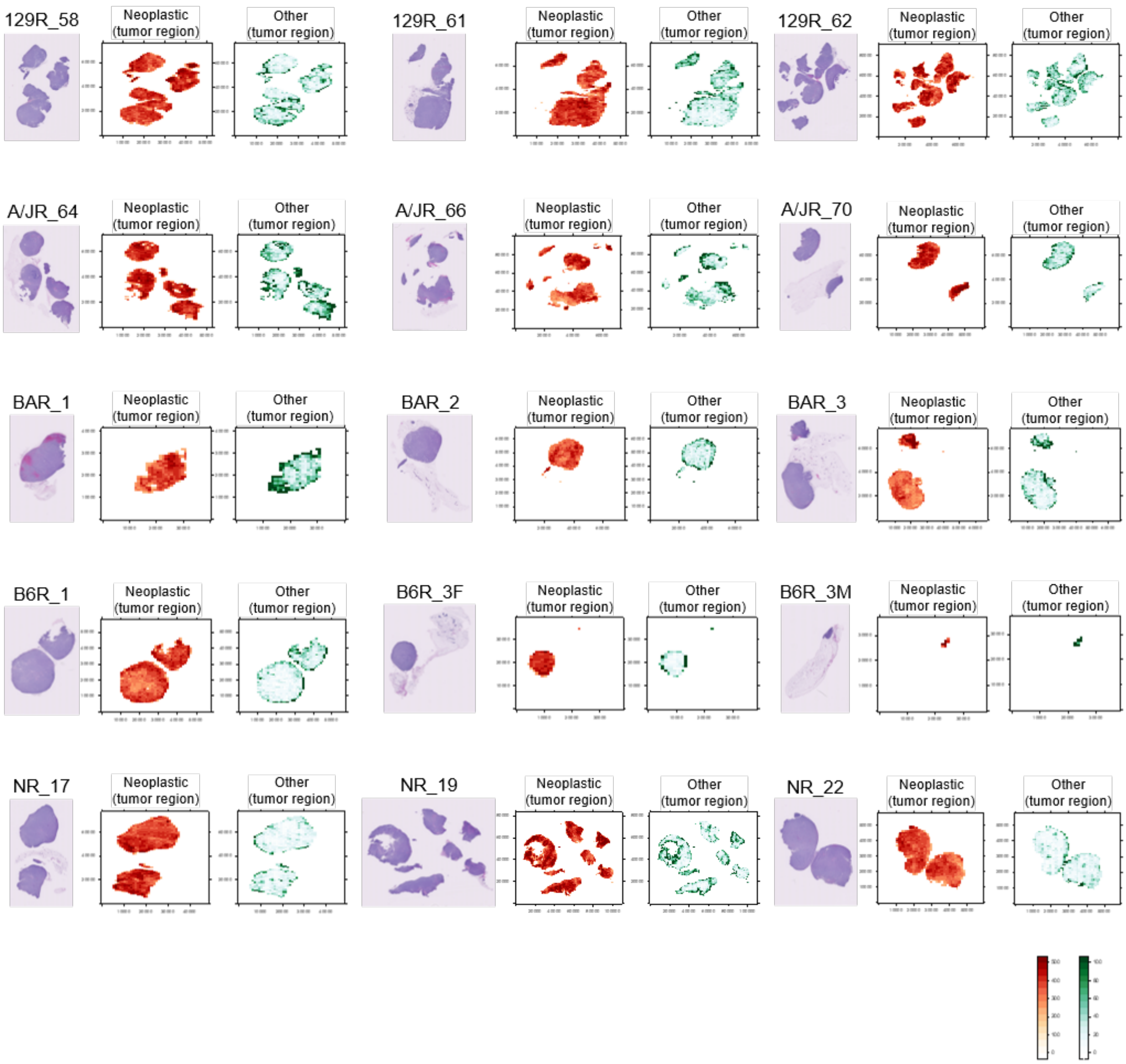

Fig. S5. H&E image and spatial distribution of neoplastic and other nuclei types for each tumor sample of the 129R, A/JR, BAR, B6R, and NR strains. The nuclei density is defined as the number of nuclei per 1024 pixel x 1024 pixel tile. The heatmaps show only tumor regions with >200 neoplastic nuclei per tile. The other nuclei types include connective, inflammatory, non-neoplastic epithelial, dead, and non-labeled nuclei.

Disease Models & Mechanisms • Supplementary information

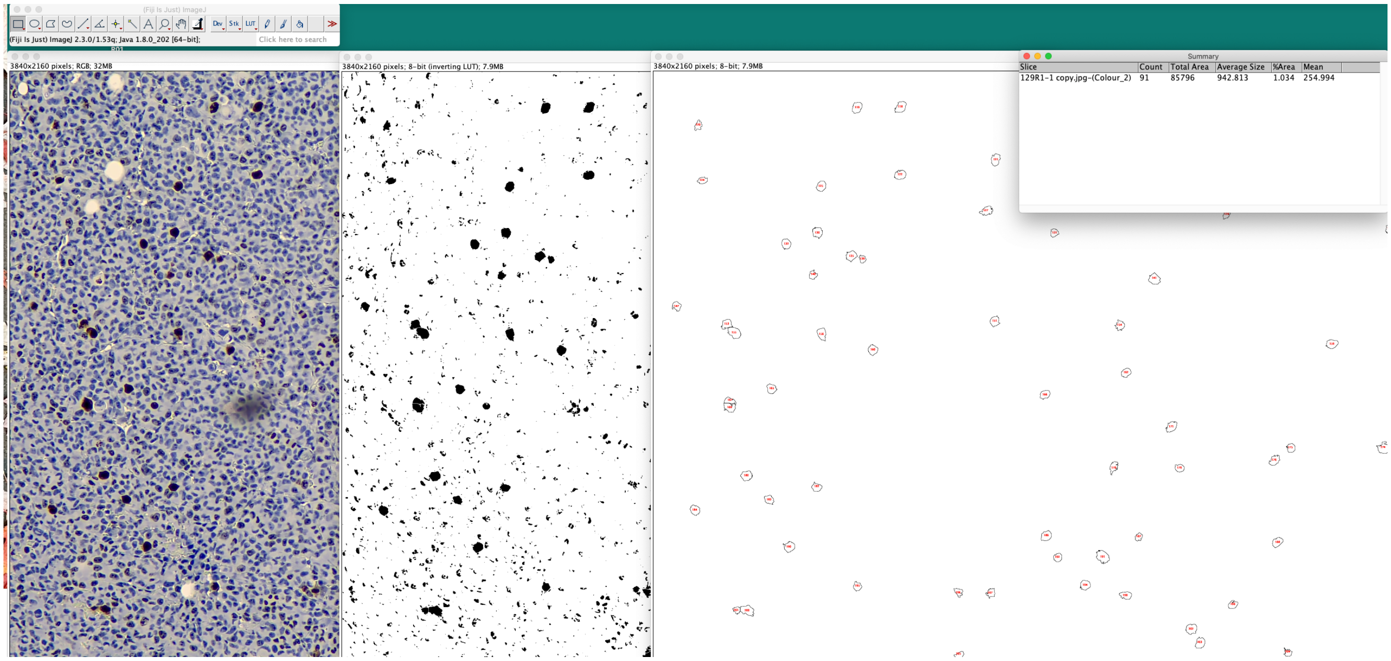

Fig. S6. Fig. S6 Method of Ki67 counting using Fiji Software. Slides were photographed at 40X and opened in Fiji Software. Process of analyzing IHC images based on https://www. ncbi. nlm.nih.gov/pmc/articles/PMC6924920/ and https://www.youtube.com/watch?v=D1qBaFwuF4E

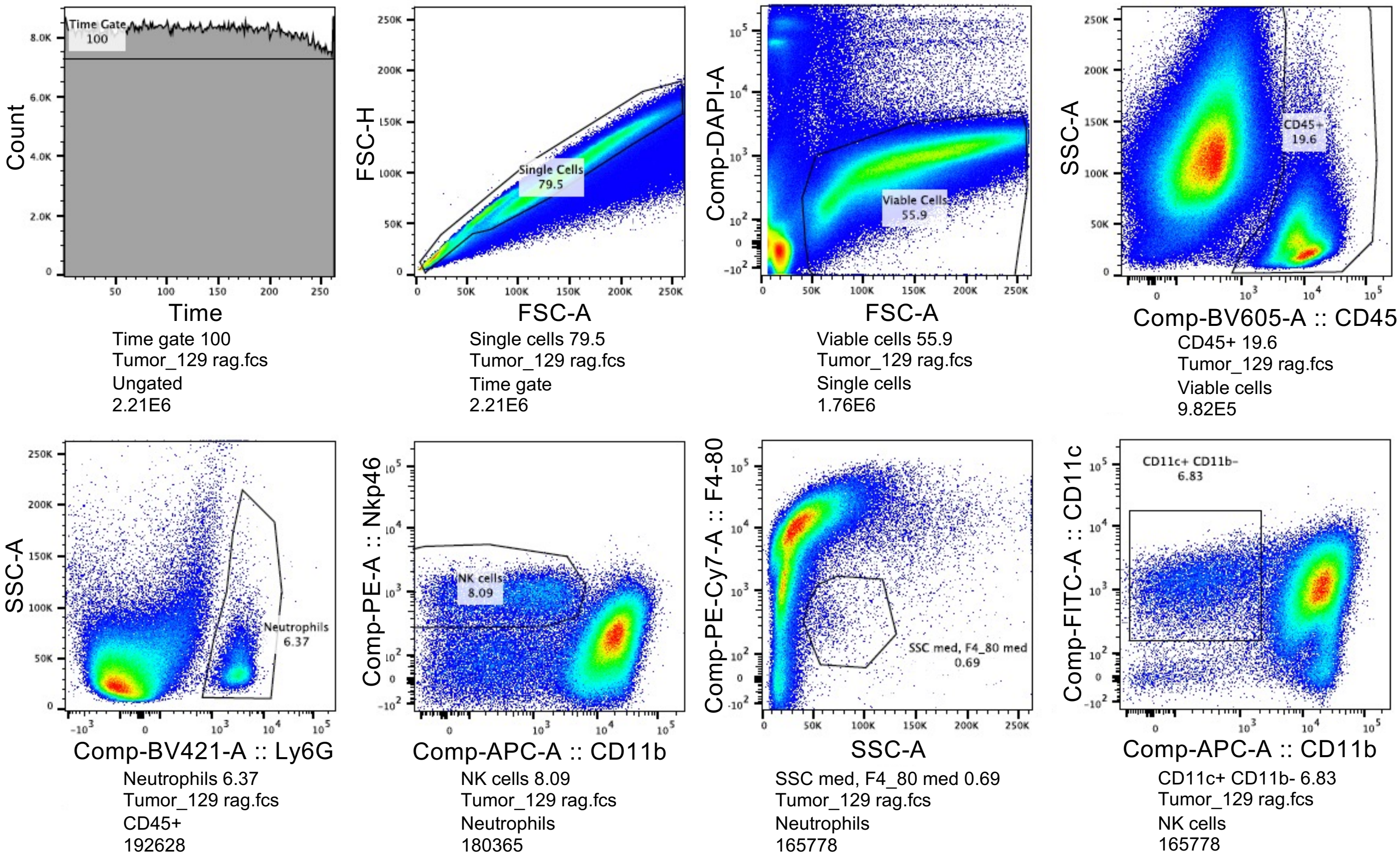

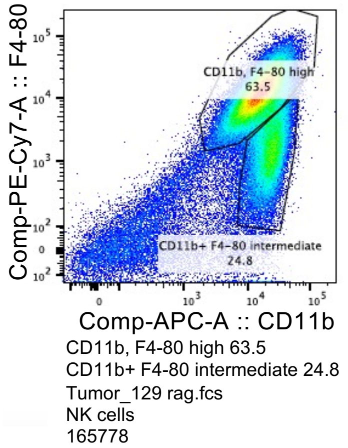

Table for figure 4C & 4D

	Neutrophil	NK	CD11b+ F4/80 int	CD11b+ F4/80 high	CD11c+ CD11b-	SSC med F4/80 med
B6 vs NOD	0.98	0.79	**0.86**	**0.69**	**0.14**	**0.08**
B6 vs 129	0.04	0.60	**0.01**	**0.04**	**0.03**	**0.90**
B6 vs A/J	0.0003	0.78	**0.0004**	**0.0003**	**0.90**	**0.00001**
B6 vs BALB	0.11	0.91	**0.28**	**0.17**	**0.66**	**0.05**
129 vs A/J	0.07	0.17	**0.87**	**0.18**	**0.03**	**0.0001**
129 vs BALB	0.74	0.32	**0.38**	**0.82**	**0.02**	**0.07**
129 vs NOD	0.03	0.69	**0.01**	**0.01**	**0.003**	**0.21**
A/J vs BALB	0.33	0.79	**0.38**	**0.21**	**0.75**	**0.11**
A/J vs NOD	0.0002	0.11	**0.0002**	**0.00001**	**0.22**	**0.000001**
NOD vs BALB	0.10	0.35	**0.28**	**0.09**	**0.44**	**0.01**

Fig. S7. Gating strategy for tumor composition and statistics for the different types of myeloid cells within the tumors of different strains. The yellow highlight shows the significant differences.

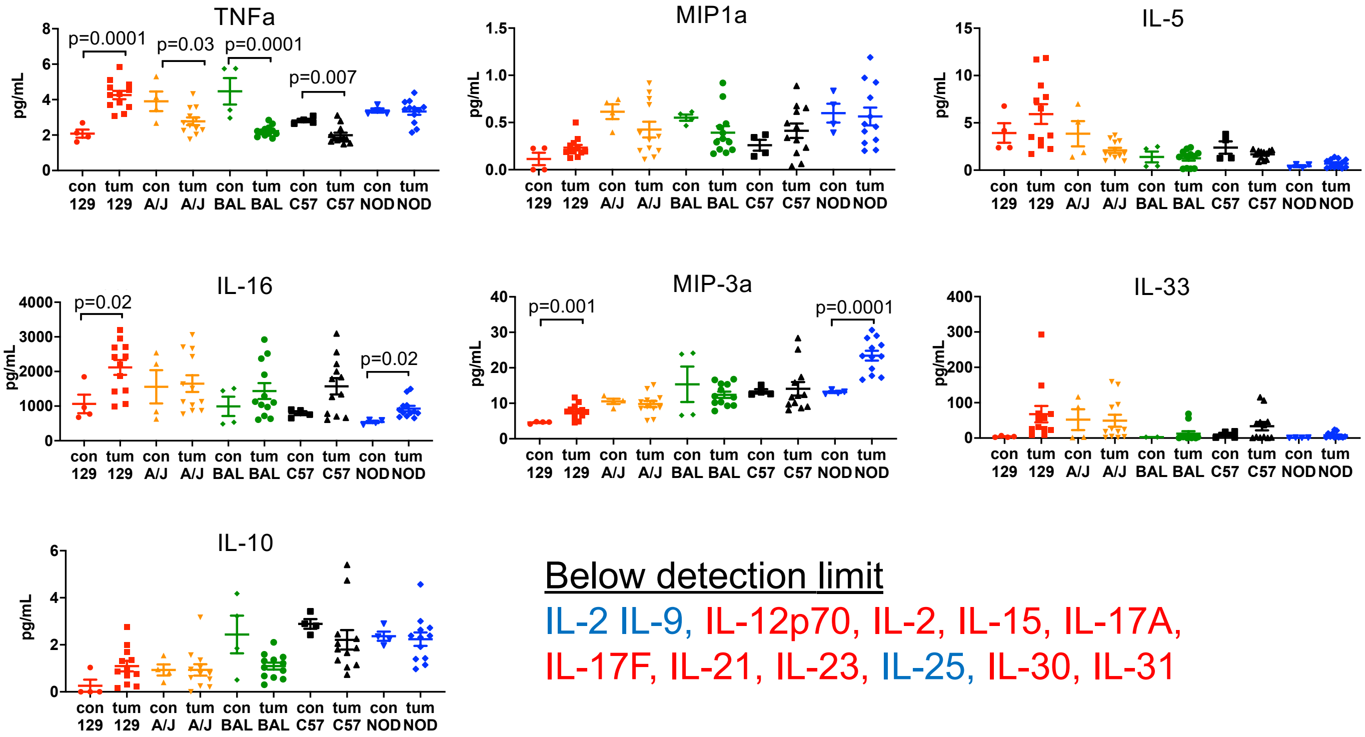

Fig. S8. Cytokines and chemokines circulating in the plasma that did not show did not show >2 fold changes or significant differences between xenografted verses non xenografted in any strain mice. Each dot represents a point of measurement, bars show mean. List of cytokines and chemokines that were below detection limit are listed.

Table S1. Fecundity of *Rag1* knockouts

strain	number of pairs mated	average mating time to pups	total number of pups	total number of litters	average litter size	pups survived to adulthood
129S1/SvImJ Rag1-/-	11, 6 still active, 2 new	31.7 days	304	62	4.9	294
A/J Rag1-/-	11, 4 still active, 2 new	54.9 days, some after ~21 days others months	100	27	3.7	94
BALB/cJ Rag1-/-	13, 7 still active, 3 new	37.5 days	194	43	4.5	191
CAST/EiJ Rag1-/-	4, 4 active, 1 new, 1 no litter	59.5 days, first 22 days other 97 days	22	8	2.8	16
C57BL/6J Rag1-/-	12, 6 still active, 2 new	35.6 days	342	58	5.9	320
DBA/J Rag1-/-	3, 0 active	56.5 days, one never produced	8	3	1.6	5
NOD/ShiLtJ Rag1-/-	14, 6 still active, 4 new	36.2 days	432	49	8.8	369
NZO/HiltJ Rag1-/-	9, 7 active, 1 new, 1 no litter	34.2 days	81	18	4.5	76
PWK/PhJ Rag1-/-	1, 1 still active	22 days	7	1	7	7
WSB/EiJ Rag1-/-	0	0	0	0	0	0
